# Downregulation of MicroRNA-130a Contributes to Endothelial Progenitor Cell Dysfunction in Diabetic Patients via Its Target Runx3

**DOI:** 10.1371/journal.pone.0068611

**Published:** 2013-07-12

**Authors:** Shu Meng, Jiatian Cao, Xiaoping Zhang, Yuqi Fan, Lu Fang, Changqian Wang, Zhongwei Lv, Da Fu, Yigang Li

**Affiliations:** 1 Department of Cardiology, Xinhua Hospital Affiliated to Shanghai Jiaotong University School of Medicine, Shanghai, China; 2 Department of Cardiology, Ninth People’s Hospital Affiliated Shanghai Jiaotong University School of Medicine, Shanghai, China; 3 Department of Nuclear Medicine, Shanghai 10th People’s Hospital, Tongji University School of Medicine, Shanghai, China; 4 The Key Laboratory of Stem Cell Biology, Institute of Health Sciences, Shanghai Institutes for Biological Sciences, Chinese Academy of Sciences/Shanghai JiaoTong University School of Medicine, Shanghai, China; 5 Vascular Pharmacology Laboratory, Baker IDI Heart and Diabetes Institute, Melbourne, Australia; University of Padova, Medical School, Italy

## Abstract

Dysfunction of endothelial progenitor cells (EPCs) contributes to diabetic vascular disease. MicroRNAs (miRs) have emerged as key regulators of diverse cellular processes including angiogenesis. We recently reported that miR-126, miR-130a, miR-21, miR-27a, and miR-27b were downregulated in EPCs from type II diabetes mellitus (DM) patients, and downregulation of miR-126 impairs EPC function. The present study further explored whether dysregulated miR-130a were also related to EPC dysfunction. EPCs were cultured from peripheral blood mononuclear cells of diabetic patients and healthy controls. Assays on EPC function (proliferation, migration, differentiation, apoptosis, and colony and tubule formation) were performed. Bioinformatics analyses were used to identify the potential targets of miR-130a in EPCs. Gene expression of miR-103a and Runx3 was measured by real-time PCR, and protein expression of Runx3, extracellular signal-regulated kinase (ERK), vascular endothelial growth factor (VEGF) and Akt was measured by Western blotting. Runx3 promoter activity was measured by luciferase reporter assay. A miR-130a inhibitor or mimic and lentiviral vectors expressing miR-130a, or Runx3, or a short hairpin RNA targeting Runx3 were transfected into EPCs to manipulate miR-130a and Runx3 levels. MiR-130a was decreased in EPCs from DM patients. Anti-miR-130a inhibited whereas miR-130a overexpression promoted EPC function. miR-130a negatively regulated Runx3 (mRNA, protein and promoter activity) in EPCs. Knockdown of Runx3 expression enhanced EPC function. MiR-130a also upregulated protein expression of ERK/VEGF and Akt in EPCs. In conclusion, miR-130a plays an important role in maintaining normal EPC function, and decreased miR-130a in EPCs from DM contributes to impaired EPC function, likely via its target Runx3 and through ERK/VEGF and Akt pathways.

## Introduction

Coronary artery disease (CAD), a leading cause of death worldwide, is largely initiated with various endothelial injuries. The endothelium has regenerative capabilities that offer protection against atherosclerosis. It is believed that the damaged endothelium can not only be repaired by the proliferation and migration of neighboring endothelial cells, but also by endothelial progenitor cells (EPCs) [1], [2]. EPCs are mobilized from bone marrow, migrate to ischemic tissue, and contribute to ischemia-induced neovascularization [3]. Therefore, EPC dysfunction may play an important role in atherosclerosis and CAD.

Diabetes mellitus (DM) is one of the most important risk factors for CAD, and CAD, in turn, is a major cause of death in patients with type II DM [4]. The loss of the modulatory role of endothelium is a critical and initiating factor in the development of diabetic vascular disease. Studies have demonstrated that DM reduces the number of EPCs and adversely affects the functional capacity of existing EPCs [5], [6], leading to a subsequent reduction in the ability of EPCs to repair the vascular endothelium [7], [8], [9]. A reduced angiogenic potential of EPCs has also been reported in diabetic animals [10]. Elucidating the basic mechanisms responsible for the diabetes-associated defects in EPC function is exceptionally important and has a high clinical impact on future interventional research.

MicroRNAs (miRs) are an emerging class of highly conserved, noncoding small RNAs that regulate gene expression at the post-transcriptional level by inhibiting protein translation or by promoting mRNA degradation [11], [12]. MiRs are transcribed by RNA polymerase II as part of a primary transcript and are degraded by the RNAse III Drosha, and DGCR8 into smaller segments of RNA [13]. Mature miRs specifically bind to 3′-UTRs of target cellular mRNAs, leading to either mRNA degradation or inhibition of translation [14]. MiRs are involved in the regulation of key cellular processes, such as proliferation [15], differentiation [16], migration [17] and apoptosis [18]. Under cell stress conditions deregulation of miRs is often observed, which may result in the development of disease, including CAD [19]. In vascular cells, miRs are important for regulating vascular signaling and function. Notably, EPCs are the prominent type of cells involved in the process of angiogenesis [20].

Our recent study has reported that miR-126, miR-21, miR-27a, miR-27b and miR-130a are downregulated in EPCs derived from type II DM patients, and downregulation of miR-126 impairs EPC function via its target, Spred-1, and through Ras/extracellular signal-regulated kinase (ERK)/vascular endothelial growth factor (VEGF) and phosphatidylinositol 3′-kinase (PI3K)/Akt/endothelial nitric oxide synthase (eNOS) signal pathway [21]. MiR-130a has been shown to play an important role in maintaining endothelial cell proliferation, migration and tubulogenic activity [22]. However, the role of miR-130a in EPC function has not been reported to date. Therefore, the aim of the present study was to investigate the contribution of dysregulated miR-130 to EPC dysfunction as well as its signaling pathways.

## Methods

The study protocol conformed to the principles outlined in the Declaration of Helsinki for the use of human blood. Written informed consent was obtained from each patient and the investigation was approved by the Ethics Committee of Experimental Research, JiaoTong University Shanghai Medical College.

### Isolation and Characterization of EPCs

EPCs were cultured as we described previously [21], [23]. PBMCs were isolated using Ficoll-Isopaque Plus (Histopaque-1077, Sigma) density gradient centrifugation of peripheral blood. Then,CD133 cells were selected from PBMCs using CD133-coupled magnetic microbeads (Miltenyi Biotech) according to the manufacturer’s instructions. CD133+ cells were then seeded into fibronectin-coated 6-well plates and cultured in endothelial basal medium (EBM, Cambrex) supplemented with VEGF (Peprotech), human recombinant long insulin-like growth factor-1, ascorbic acid, cortisol, and 20% fetal calf serum at 37°C in a 5% CO_2_ incubator. After 4 days, medium was refreshed and after 7 days of culture, early EPCs developed an elongated spindle-shaped morphology. Phenotypic characterization of early EPCs was performed by confocal microscopy and flow cytometry. The cells were incubated with agglutinin 1 (FITC-UEA-1; Sigma Deisenhofen, Germany) and 1, 19-dioctadecyl-3, 3, 3939-tetramethylindocar- bocyanine perchlorate (DiI)-labeled acetylated low density lipoprotein (Dil-Ac-LDL) as previously described [24]. Incorporation of DiI-Ac-LDL and binding of FITC-UEA-1 were detected with a confocal microscope (Leica Microsystems GmbH). Cells positive for dual-staining of DiI-Ac-LDL and UEA-1 were identified as EPCs. The purity of EPCs was analyzed by flow cytometry after staining with anti-CD34, anti-KDR, anti-CD133, anti-CD14 and anti-CD45 (all antibodies conjugated with PE, from BD). EPCs after 7 days of culture were used for flow cytometry analysis of EPC apoptosis and differentiation, Proliferation assay, Migration assay, Tubule formation assay. The characteristics of EPC donors are described in Table S1. EPCs from 5 donors were pooled for real-time PCR and western blotting.

### Real-time PCR

Total RNA was isolated from EPCs using Trizol reagent (Invitrogen) according to the manufacturer’s instruction. RNA was reverse transcribed using the stem-loop RT primer as previously described [25]. Real-time PCR was performed using a Roche LightCycler 480 system. All reactions were performed in triplicates. U6 and β-actin were used as endogenous controls.

### Western Blotting

Western blotting was performed as described previously [26]. In brief, EPCs were lysed with 100 mM phenylmethanesulfonyl fluoride, and the protein extracts were denatured and loaded onto a 10% SDS-PAGE gel. The separated proteins were transferred to a PVDF membrane and probed with primary antibodies including anti-Runx3, anti-β-actin, anti-Akt1, anti-p-ERK, anti-VEGF antibody (Santa Cruz Biotechnology), anti-PI3K antibody (Cell Signaling Technology, Inc) and the protein signals were detected using Odyssey (Li-cor, USA) and semi-quantitatively analysis was performed by using Quantity one (Bio-Rad).

### Anti-miR-130a Transfection

To inhibit miR-130a, a miR-130a inhibitor (Sigma-Aldrich) or a negative control was transfected into EPCs using lipofectamine 2000 (Invitrogen) according to the manufacturer’s instructions [26]. Cells were harvested 36 h after transfection, and miR-130a expression was analyzed by real-time PCR.

### Lentiviral Constructs, Packaging, and Transduction

Expression plasmids for miR-130a and Runx3 were created using PCR amplification with human genomic DNA as templates. The primers are described in Table 1. The PCR product of miR-130a was cloned into PLKO.1-puro vector (Sigma-Aldrich). The PCR product of Runx3 was cloned into lentiviral expression plasmid pCDH-EF1-MCS-T2A-Puro (System Biosciences, SBI, CD520A-1), while a cDNA encoding a short hairpin RNA (shRNA) targeting Runx3 (Table 1) was cloned into the PLKO.1-shRNA1 vector. All constructs were confirmed by sequencing. To produce lentivirus, these three plasmid DNAs were individually transfected into 293 T cells using psPAX2, a pMD2G packaging construct, and lipofectamine plus reagent (Invitrogen) according to the manufacturer’s protocol. After 6 h, the medium was refreshed, and viral supernatant was collected 48 h later. EPCs were seeded into 6-well plates (5×10^5^ cells per well) and cells were transfected with different lentiviral vectors at an MOI of 10 as previously described [25]. 48 h after infection, cells were selected and cloned by culture in the presence of puromycin (2 µg/mL) for 1 week.

**Table 1:** Oligonucleotides for cloning miR-130a, shRNA and 3′UTRs of target genes.

	Primer	Sequence
**A**	miR-130a	F5′- AAAGGATCCGCATCAAGCCCGAAGTAT -3′
		R5′- AAAGAATTCGAGGCAGTGTCTATCACAAG -3′
**B**	Runx3	F 5′-GGCGTAAGGGAACTCATAAAG-3′
		R 5′-GGAGGGAAGAAACTACAAGGAC -3′
**C**	Runx3shRNA	F 5′- CCGGGGCTAGCAGCATGCGGTATTTCTCGAGAAATACCGCATGCTGCTAGCCTTTTTG-3′
		R 5′- AATTCAAAAGGCTAGCAGCATGCGGTATTTCTCGAGAAATACCGCATGCTGCTAGCC-3′
**D**	Runx3UTR	F 5′-CCGCCCTGGTGGACTCCT-3′
		R 5′-CCTTCCACACATCTCAGAGTTATAT-3′

### DNA Constructs and Report Gene Assay

For luciferase reporter experiments, the 3′UTR of the Runx3 gene was amplified by PCR from human genomic DNA and cloned into the psiCHECK™-2 vector (Promega) between the Not1 and Sgf1 sites. The primers are described in Table 1. The construct with mutated targeting fragment (TGGCGCGCC) at the 3′UTR of RUNX3 without the putative miR-130a binding sequence was used as a mutated control. EPCs were seeded into 24-well plates (1×10^5^ cells/well) by using lipofectamine 2000 according to the manufacturer’s protocol. Cells treated with 10 nM miR-130a inhibitor or negative control, or with 10 nM miR-130a mimic or mimic negative control (Sigma-Aldrich) were transfected with 0.1 µg of the p-Runx3 UTR firefly luciferase report vector and 0.02 µg pRL-TK (Promega) for normalization of transfection [27]. After 48 h, cells were washed and lysed with passive lysis buffer, and firefly luciferase activity was determined using the dual-luciferase reporter assay system and a luminometer (Promega) as we previously described [27]. The relative reporter activity was obtained by normalizing the firefly luciferase activity against the internal control luciferase activity.

### Flow Cytometry Analysis of EPC Apoptosis and Differentiation

Apoptosis, and differentiation of EPCs were measured using flow cytometric analysis [28]. Apoptotic cells were measured by dual labeling with anti-annexin V (eBioscience, San Diego) and propidium iodide (eBioscience, San Diego). Cells were incubated with PE-conjugated CD64 (BD Biosciences) and FITC-conjugated anti-von Willebrand factor (vWF, BD Biosciences) to measure EPC differentiation.

### Colony Formation Assay

EPC colony-forming capacity was determined as previously described [25]. Isolated PBMCs were resuspended in EBM medium, and 5×10^6^ PBMCs were seeded into fibronectin-coated 6-well plates. After two days, non-adherent cells were collected and 1×10^6^ cells were replated into 24-well fibronectin-coated plates. On the 5^th^ day, EPC colony-forming units were manually counted in 4 random wells by 2 independent, blinded investigators. Only clusters consisting of a central core of rounded cells surrounded by elongated and spindle-shaped cells were counted.

### Proliferation Assay

Proliferation of EPCs was evaluated by bromodeoxyuridine (BrdUrd) incorporation as described previously [29]. Briefly, cells were cultured in EBM medium following 24 h of serum starvation. After 18 h in culture, BrdUrd (10 µmol/L, Sigma) was added to each well and incubated with cells for 72 h. BrdUrd was visualized by immunostaining with anti-BrdUrd antibody (1∶100, Biodesign), and proliferation activity was expressed as mean percentage of BrdUrd positive cells counted in 5 high-power fields (400×) in each well.

### Migration Assay

Migration assays were performed in a modified Boyden chamber (NeuroProbe) [25], [30]. EPCs were resuspended in serum-free EBM medium, and 4×10^4^ cells were loaded into the upper chambers. The lower chamber was filled with EBM medium containing VEGF (50 ng/mL). After 24 h of incubation at 37°C, cells were stained with hematoxylin and counted in 3 random fields (200×) in each well. All groups were performed in triplicate.

### Tubule Formation Assay

In vitro neovascularization assays were performed in human fibrin matrices as described previously. [24] In brief, EPCs transfected with lentivirus, and then serum-starved EPCs were seeded onto matrigel (BD Bioscience)-coated plate in EBM medium and incubated at 37 for 24 h, tubular structures of EPCs in the matrigel were analyzed by phase-contrast microscopy. To quantify the length of newly formed tubes, 6 random phase-contrast photomicrographs per well were taken, and the length of each tube was measured using Quantity one Image software. Tube length obtained from miR-130a transfected cells was set to 100.

### Statistical Analysis

Data were expressed as mean±SD. Student’s t-test was used to compare differences between 2 groups. One-way ANOVA followed by tukey’s post-hoc tests or two-way ANOVA were used to compare differences among multiple groups when appropriate. A value of *P*<0.05 (two-sided) was considered statistically significant. All experiments were performed at least 3 times.

## Results

### Characterization of EPCs and Downregulation of miR-130a in Diabetic EPCs

**Figure 1:**
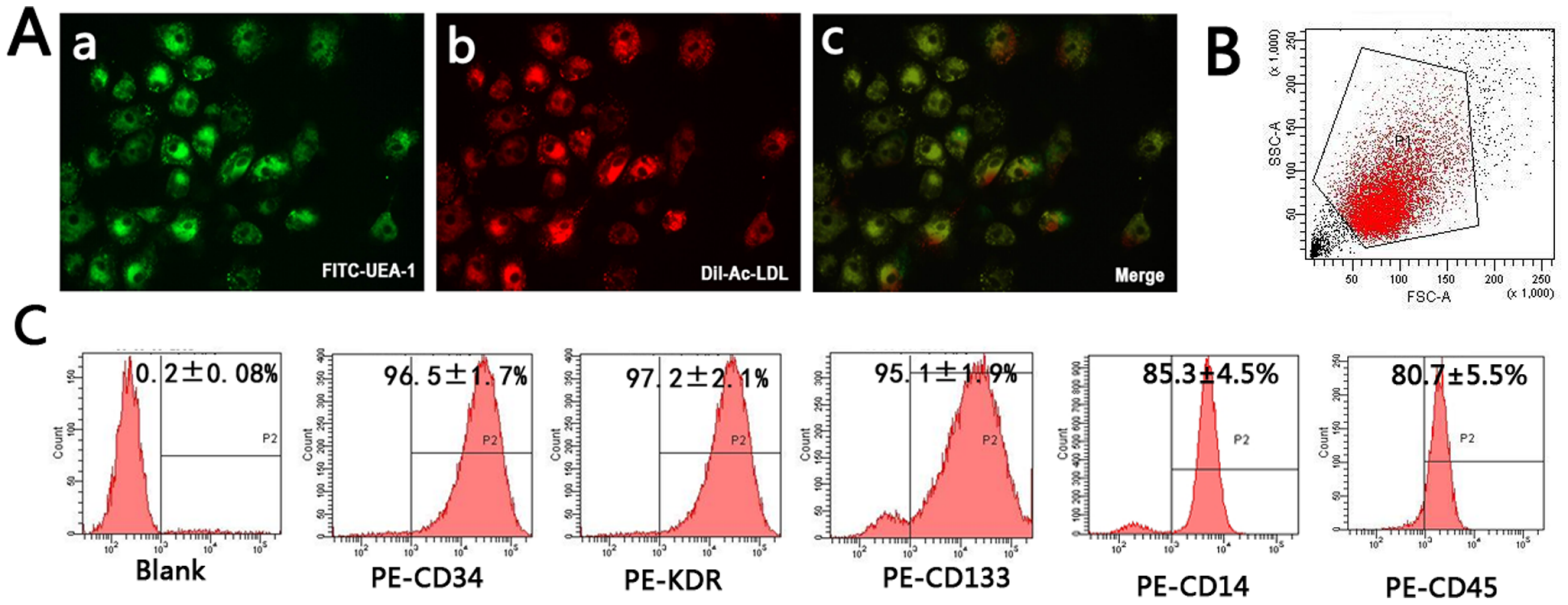
Characterization of EPCs by confocal microscopy and flow cytometric analysis. (A) Staining of FITC-UEA-1 (a), DiI-Ac-LDL (b), and dual staining of FITC-UEA-1 and DiI-Ac-LDL (c) were detected using a confocal microscope. (B) A representative FSC/SSC plot. (C) Flow cytometric analysis of the cell surface markers of EPCs (CD34, KDR, CD133, CD14 and CD45). The presented experiment is a typical result obtained from three separate experiments.

Early EPCs after 7 days of culture developed an elongated spindle-shaped morphology. EPCs were then characterized by double-positive staining with FITC-UEA-1 and DiI-Ac-LDL (Fig. 1A). These EPCs also expressed high levels of EPC markers such as CD34, CD133, KDR, CD14 and CD45 (Fig. 1C). In our previous study, we reported that miR-130a was downregulated in EPCs derived from patients with diabetes [25], suggesting that the low level of miR-130a in diabetic EPCs may be involved in functional impairment of EPCs in these patients.

### MiR-130a Inhibition Induced Runx3 Gene and Protein Expression in EPCs

**Figure 2:**
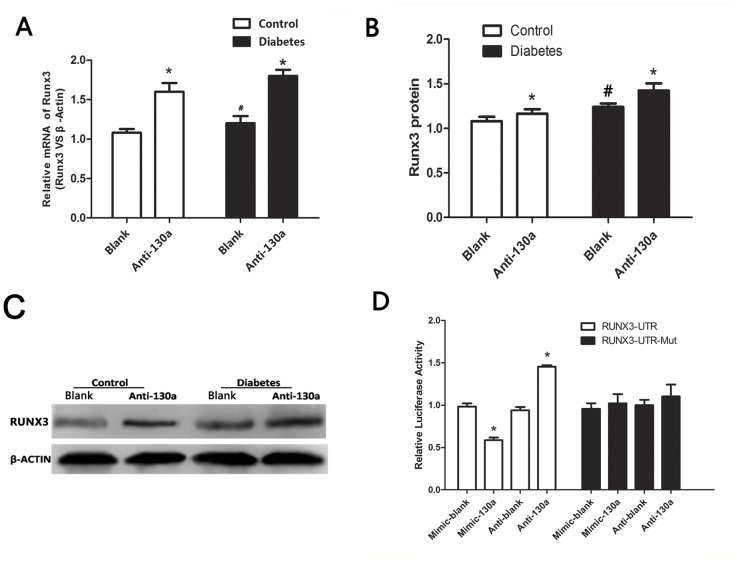
MiR-130a suppressed Runx3 expression. (A–C) Cells were transfected with a miR-130a inhibitor (anti-miR-130a) or a negative control (blank). (A) Runx3 mRNA level measured by real-time PCR (normalized to β-actin), (B–C) Protein expression of Runx3 by Western blotting and respective densitometric measurement results of Runx3, (D) Luciferase reporter assay. The partially complementary miR-130a-binding site found in the Runx3 3′-UTR (or a mutated binding site) was inserted downstream of a luciferase reporter on the psiCHECK™-2 Vector report plasmid and transfected into EPCs with miR-130a mimic or miR-130a inhibitor. *P<0.05 vs. respective scrambled control (blank) groups. #*P*<0.05 vs. healthy control group. Similar results were obtained in at least three separate experiments and error bars represent the standard deviation from the mean.

We searched potential targets of miR-130a from multiple databases (Targetscan, PicTar, and miRanda) and proposed that Runx3 may be a potential target of miR-130a. To evaluate this putative interaction, we first demonstrated an inverse correlation between the expression level of miR-130a and the mRNA and protein level of Runx3 in EPCs from both healthy and diabetic donors. Anti-miR-130a significantly induced Runx3 mRNA (Fig. 2A) and protein levels (relatively moderately) in both diabetic and healthy groups (Fig. 2B, 2C).

### Runx3 is a Direct Target of miR-130a in EPCs

Next, a dual luciferase reporter gene assay was performed to further examine whether Runx3 is a direct target of miR-130a. A mimic and an inhibitor of miR-130a were used to modulate miR-130a levels in EPCs. The miR-130a mimic inhibited whereas the miR-130a inhibitor increased luciferase reporter activity with a wild-type sequence (Fig. 2D). However, this effect was not observed for the mutants (Fig. 2D). This result confirms that Runx3 is a direct target of miR-130a in EPCs.

### Inhibition of miR-130a Impairs EPC Function

**Figure 3:**
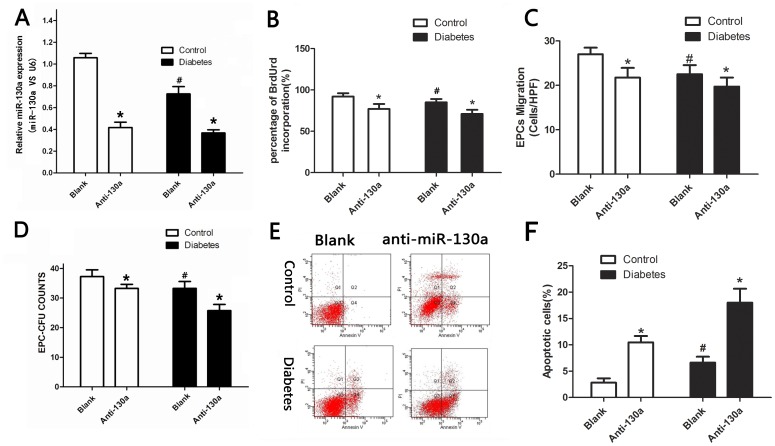
MiR-130a inhibition decreased EPC proliferation, migration and colony formation, but increased EPC apoptosis. Cells were transfected with a miR-130a inhibitor (anti-miR-130a) or a negative control (blank) (A) Gene expression of miR-130 by real-time PCR (normalized to U6). (B to F) Functional assays of EPCs: proliferation (B), migration (C), colony formation (D), and apoptosis (E, F) of EPCs. *P<0.05 vs. respective scrambled control (blank) groups. #*P*<0.05 vs. healthy control group. CFU: colony-forming units. Similar results were obtained in at least three separate experiments and error bars represent the standard deviation from the mean.

**Figure 4:**
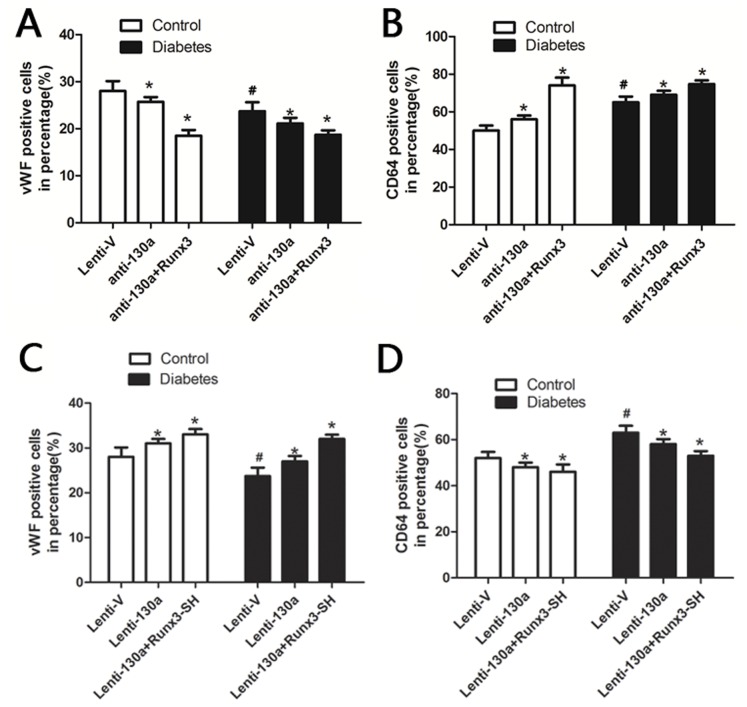
The effect of miR-130 and Runx3 on EPC differentiation. Expression of CD64 and vWF were analyzed by flow cytometry. (A, B) Cells were transfected with a miR-130 inhibitor or both miR-130 inhibitor and lentiviral Runx3. (C, D) cells were transfected with lentiviral miR-130 or both lentiviral miR-130 and lentiviral vector expressing Runx3 shRNA (Runx3-SH). Lenti-V: cells were tranfected with an empty vector. *P<0.05 vs. respective Lenti-V groups. #*P*<0.05 vs. healthy control group. Similar results were obtained in at least three separate experiments and error bars represent the standard deviation from the mean.

To investigate the role of miR-130a in EPC function, we inhibited endogenous miR-130a in EPCs by transfecting EPCs with a miR-130a inhibitor or a negative control. We first confirmed that miR-130a gene expression was decreased by the miR-130 inhibitor in both healthy and diabetic groups (Fig. 3A). We then found that inhibition of miR-130 adversely affected EPC function. Anti-miR-130a significantly reduced proliferation, migration and colony formation of EPCs (Fig. 3B–D), but increased apoptosis of EPCs (Fig. 3E, 3F) compared to the negative control. It is known that when EPCs differentiate into endothelium cells, vWF expression increases, whereas CD64 expression decreases [25]. We found that vWF expression decreased, but CD64 expression increased in anti-miR-130a transfected EPCs in both the diabetic and control groups (Fig. 4A, 4B), suggesting that anti-miR-130a inhibits EPC differentiation. These results suggest that miR-130a is fundamental to maintain normal EPC function.

### MiR-130a Modulated EPCs Function by Targeting Runx3

**Figure 5:**
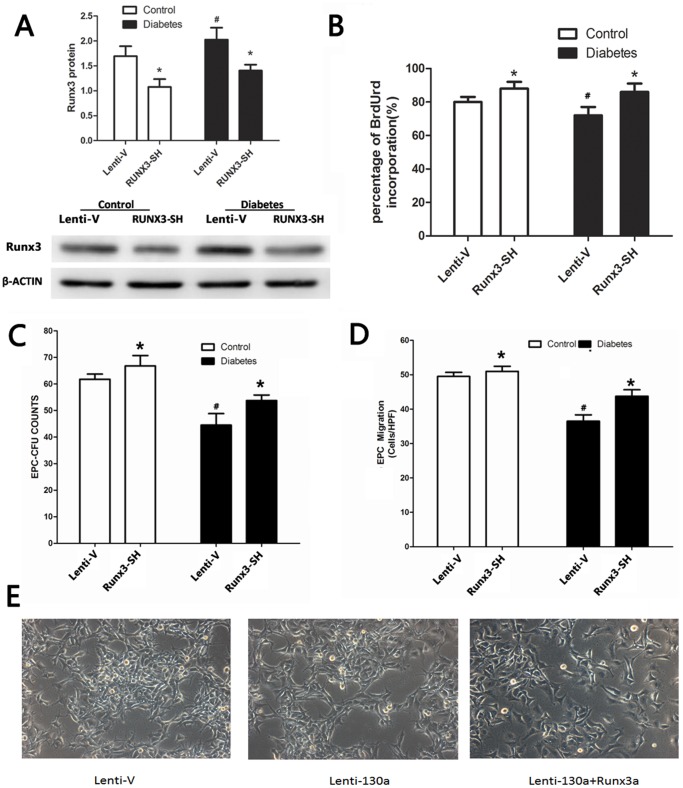
Runx3 repression promoted EPC function. (A–D) Cells were transfected with a lentiviral vector expressing Runx3 shRNA to downregulate Runx3 expression (Runx3-SH) or an empty vector (Lenti-V). (A) Runx3 protein levels by Western blotting and densitometric measurement results of Runx3 protein. (B) EPC proliferation assay. (C) EPC colony formation assay (D) Migration assay. (E) Cells were transfected with a lentiviral vector expressing miR-130a or both miR-130a and Runx3. Tubule formation assay was performed. *P<0.05 vs. respective Lenti-V groups.^ #^ *P*<0.05 vs. healthy control groups for A–D. Similar results were obtained in at least three separate experiments and error bars represent the standard deviation from the mean.

**Figure 6:**
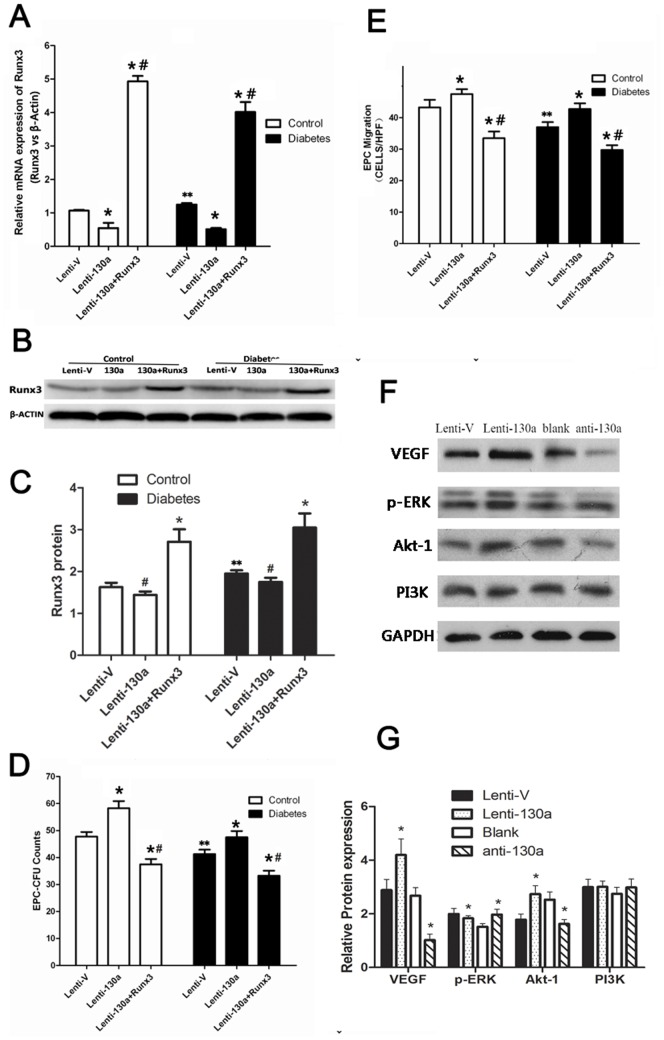
Runx3 mediated miR-130a’s effect on EPC function and miR-130a upregulated VEGF, p-ERK and Akt1. (A–E) Cells were transfected with both lentiviral miR-130a and lentiviral Runx3 or lentiviral miR-130a alone. (A) mRNA expression of Runx3 by real-time PCR (normalized to β-actin). (B, C) Protein expression of Runx3 by Western blotting. (D, E) Colony formation and migration capacity of EPCs. * *P*<0.05 vs. respective empty vector groups (Lenti-V). # *P*<0.05 vs. lenti-130a group. ** *P*<0.05 between the same processed healthy and diabetic groups (eg. Lenti-130a control groups VS Lenti-130a diabetes groups ) (F, G) EPCs were transfected with a miR-130a inhibitor or a negative control (Blank), or lentiviral miR-130 or an empty vector (Lenti-V). Protein levels of VEGF, p-ERK and Akt1 were measured by Western blotting. *P<0.05 Lenti-130a vs. Lenti-V group or anti-130a vs. scrambled control (blank) group. VEGF: vascular endothelial growth factor; ERK: extracellular signal-regulated kinase; PI3K: phosphatidylinositol 3′-kinase. The presented experiment is a typical result obtained from three separate experiments.

To explore the role of Runx3 in EPC function, EPCs were transfected with a lentiviral vector expressing Runx3 shRNA. Transfection of Runx3 shRNA reduced protein level of Runx3 expression in EPCs in both diabetic and healthy subjects (Fig. 5A). EPCs transfected with Runx3 shRNA showed increased cell proliferation, colony formation, migration (Fig. 5B–D) as compared to cells transfected with empty vector controls. We further explored whether miR-130a mediated EPC function by regulating the expression level of Runx3. We constructed lentiviral vectors expressing Runx3 and lenti-miR130a to transfect EPCs. Lenti-miR-130a alone downregulated expression levels of Runx3 (Fig. 6A), promoted colony formation (Fig. 6D), migration (Fig. 6E), differentiation (Fig. 4C, Fig. 4D) and EPCs tube formation (Fig. 5E, 5F) compared to empty vector control. The cells infected with both lenti-Runx3 and lenti-miR-130a upregulated mRNA and protein expression levels of Runx3 (Fig. 6A–C), but decreased EPC colony formation and migration capacity (Fig. 6D, 6E) and tubule formation (Fig. 5E, 5F) compared to EPCs transfected with lenti-miR130a alone. We also studied whether miR-130a affected PI3K/Akt and ERK/VEGF pathways. Lenti-miR-130a upregulated while anti-miR-130a downregulated the protein expression of VEGF, p-ERK and Akt1 in EPCs (Fig. 6F, 6G). These data confirm that Runx3 is downregulated by miR-130a and also indicate that repression of Rux3 by miR-130a mediates the role of miR-130a in maintaining normal function of EPCs. In addition, miR-130a may exert its effect on EPCs through ERK/VEGF and Akt pathway.

## Discussion

In this study we have made several important findings. First, miR-130a was decreased in EPCs derived from patients with type II DM. Inhibition of miR-130a decreased proliferation, migration, differentiation, colony formation, and angiogenic potential of EPCs, but increased apoptosis of EPCs. Next, we found that Runx3 is a target of miR-130a in EPCs. Runx3 negatively regulated EPC function and repression of Runx3 by miR-130a mediated miR-130’s effect on EPC function. Furthermore, miR-130a regulated ERK/VEGF and Akt1 in a positive way. Taken together, our findings suggest that miR-130a plays an important role in maintaining normal EPC function, and decreased miR-130a in EPCs from DM contributes to impaired EPC function, which is likely due to increased Runx3 and decreased VEGF.

A strong correlation exists between cardiovascular risk factors and the number and function of EPCs [23]. DM has also been shown to adversely affect the number and function of EPCs [5], [6], leading to a decreased ability of EPCs to perform endothelial repair. Accumulating evidence suggests that miRs are involved in the process of angiogenesis by modulating new vessel formation [31], [32]. Overexpression of miR-221 and miR-222 [33] in human vascular endothelial cells significantly reduced endothelial cell migration, proliferation and angiogenesis *in vitro*, whereas miR-130a, miR-210, miR-424, miR-27-b, let-7f, and the miR-17-92 cluster were identified as proangiogenic miRs [34]. Our recent study has detected downregulation of miR-126, miR-21, miR-27a, miR-27b and miR-130a in EPCs derived from type II DM patients. We further demonstrated that downregulation of miR-126 impairs EPC function via its target, Spred-1, and through Ras/ERK/VEGF and PI3K/Akt/eNOS signal pathway [25]. Since miR-130a has been found to play a pivotal role in maintaining endothelial cell proliferation, migration and tubulogenic activity [22], in the present study, we investigated whether downregulation of miR-130a were also associated with impaired EPC function in type II DM. We found that interfering with miR-130a in EPCs inhibited proliferation, migration, differentiation and colony formation (but increased apoptosis), while overexpression of miR-130a in EPCs promoted migration, differentiation, colony formation, and tubule formation. So, reduced level of miR-130a contributes to EPC dysfunction in type II DM. Administration of miR-130a may restore the ability of EPCs in diabetes to incorporate into the damaged endothelium and work in concert with existing endothelial cells to form blood vessels. Further studies are required to examine whether other miRs such as miR-21, miR-27a and miR-27b which are decreased in DM are also related to EPC dysfunction in type II DM, and how these miRs interact with each other to maintain normal EPC function.

MiR-130a appears to be widely expressed in diverse cell types and regulate various cell function through different targets in different cell types [22], [35], [36], [37], [38]]. We searched potential targets of miR-130a from multiple databases and proposed that Runx3 may be a target of miR-130a in EPCs. We demonstrated that inhibition of miR-130a increased while overexpression of miR-130a decreased Runx3 mRNA and protein levels in EPCs. Consistent with this, knockdown of Runx3 expression in EPCs derived from patients with DM promotes colony formation, proliferation, migration and differentiation of EPCs, thus rescuing EPC dysfunction due to miR-130a deficiency in DM.

The human Runx gene encodes the α-subunit of the runt domain transcription factor PEBP2/CBF and is a homolog of the Drosphila genes Runt and Lozenge. The mammalian and Drosphila Runx genes share an evolutionarily conserved region of 128 amino acids, termed the runt domain, required for DNA binding and heterodimerization with the β-subunit PEBP2/CBF. All three runt domain family members, Runx1, Runx2, and Runx3, are master regulators of gene expression in humans [39], [40], [41]. Runx3 is reported as a tumor suppressor gene for gastric cancer [42], and may be important in the development of hepatocellular carcinoma [43]. Although there are no reports on relationships between Runx3 and diabetes, or between Runx3 and EPCs, our study shows that Runx3 is expressed by EPCs, and increased Runx3 in EPCs of type II DM patients due to downregulated miR-130a causes EPC dysfunction. Further work is needed to study whether Runx-1 and Runx-2 are also targets of miR-130a.

Previous evidence has suggested a negative relationship between Runx3 and VEGF. It was found that dramatic loss of Runx3 protein was associated with VEGF overexpression and increased microvessel density in human gastric cancer. Restoration of Runx3 expression significantly inhibited gastric cancer cell growth *in vitro* and tumorigenicity and metastasis in animal models and correlated with transcriptional repression of VEGF expression [42]. It is therefore suggested that the VEGF pathway may be involved in miR-130a deficiency-induced EPC dysfunction in type II DM patients. In the present study, we have found that miR-130 negatively regulate Runx3, but positively regulate ERK/VEGF and Akt. So, downregulated miR-130a in EPCs from DM patients decreases VEGF expression and VEGF-induced angiogenesis, likely via increased Runx3.

There are currently no suitable soluble biomarkers for atherosclerosis and/or endothelial dysfunction in patients with diabetes. Inflammatory markers such as high-sensitivity C-reactive protein are widely used, but they lack specificity for the vasculature pathology. Moreover, the existing imaging techniques are capable of quantifying the presence of plaques, but imaging techniques are not able to identify early stages of vasculature pathology, particularly endothelial dysfunction. If miR-130a is uniquely modified by diabetic vascular injury, it may be capable of adding to the predictive value of conventional risk factors.

This study has some limitations. From database search, we proposed that Runx3 was a potential target of miR-130a in EPCs. Our results confirmed that Runx3 is a target of miR-130a and repression of Rux3 by miR-130a mediates the role of miR-130a in maintaining normal EPC function. However, this does not necessarily mean that Runx3 is the most important target of miR-130a in EPCs. Runx1, Runx2, and other miR-130a targets identified in other cell types should be investigated as well. In addition, our current data did not provide direct evidence on the interaction between Runx3 and VEGF in EPCs. Future studies are required to investigate how Runx3 regulates VEGF in EPCs. Up to now, various criteria for EPC selection have been used, including CD34(+) DiI-acLDL(+) lectin(+),CD34(+),CD133(+)KDR(+),CD34(−) CD14(+),CD31(+), CD14(+), CD34(+) and so on. In the present study, PBMCs derived CD133+ cells were isolated, endocytose DiI-acLDL, and bind Ulex-lectin were used as EPCs, the cells also showed high expression of KDR and CD34. EPCs have been shown to proliferate, differentiate into ECs, and form tube-like structures in vitro. The cells have the feature of progenitor cells, and they have different physiological implications in paracrine cells of the innate immune response than in endothelial cells. In consideration of this, the present result can be said to have been derived from this population of EPCs; the PBMCs derived CD133+ cells.

Taken together, this study shows that miR-130a is downregulated in EPCs from diabetic patients, which impairs EPC function via its target, Runx3, and through ERK/VEGF and Akt pathway. Future animal studies need to be conducted to explore miRs-based therapeutic interventions on vascular complications of DM.

## Supporting Information

Table S1 Baseline characteristics of study subjects.
(DOC)
