# Supplementary material for: Downregulation of MicroRNA-130a Contributes to Endothelial Progenitor Cell Dysfunction in Diabetic Patients via Its Target Runx3
Source: PLoS One. 2013 Jul 12;8(7):e68611. doi: 10.1371/journal.pone.0068611 (PMC3709913; doi:10.1371/journal.pone.0068611)
Supplement: Table S1 — Baseline characteristics of study subjects. (DOC) [file pone.0068611.s001.doc]

Supplement table. Baseline characteristics of study subjects

|  | Control  (*n* = 20) | Diabetes  (*n* = 20) |
| --- | --- | --- |
| Age, y | 62 ± 9 | 64 ± 8 |
| Body mass index, kg/m^2^ | 26 ± 9 | 28 ± 7 |
| Sex, females/males | 10/10 | 10/10 |
| Smoking, % | 15 | 20 |
| High blood pressure, % | 45 | 50 |
| Systolic blood pressure (mmHg) | 133 ±7 | 131±9 |
| Diastolic blood pressure (mmHg) | 75±6 | 78±4 |
| Hyperlipidemia, % | 30 | 25 |
| Total cholesterol (mmol/L) | 4.37±0.71 | 4.45±0.62 |
| Triglycerides (mmol/L) | 1.9±0.41 | 2.12±0.39 |
| High-density lipoprotein–cholesterol (mmolL) | 1.15±0.32 | 1.21±0.28 |
| Low density lipoprotein–cholesterol (mmolL) | 2.39±0.50 | 2.44±0.61 |
| Fasting plasma glucose (mmol/L) | 5.4±0.8 | 7.8±0.74* |
| Coronary heart disease, % | 45 | 55 |
| Insulin use, % | 0 | 30* |
| Oral pioglitazone use, % | 0 | 35* |
| ACEI/ARB use, (%) | 30 | 35 |
| Statin use, (%) | 25 | 25 |

Data are presented as mean ± SD

* *P*<0.05) vs. control group. Wilcoxon rank-sum test was used for distributed continuous variables while Fisher’s exact test was used for categorical covariates. ACEI: Angiotensin-converting enzyme inhibitors; ARB: angiotensin II receptor antagonists.
